# Insights into perceptual learning

**DOI:** 10.7554/eLife.111673

**Published:** 2026-05-21

**Authors:** Zhong-Lin Lu

**Affiliations:** 1 Division of Arts and Sciences, New York University Shanghai Shanghai China; 2 Center for Neural Science and the Department of Psychology, New York University New York United States; 3 Institute of Brain and Cognitive Science, New York University–East China Normal University Shanghai China

**Keywords:** perceptual learning, serial dependence, selective reweighting, learning generalization, visual perception, Human

## Abstract

A form of short-term memory called serial dependence can predict how effectively perceptual learning transfers to novel visual locations.

**Related research article** Pinchuk-Yacobi N, Sagi D, Bonneh YS. 2026. Serial dependence predicts generalization in perceptual learning. *eLife* **15**:RP109830. doi: 10.7554/eLife.109830.

Practice makes perfect in many areas of life – including our visual system. When people repeatedly perform a visual task, such as identifying patterns or shapes, they typically become better at it over time (Dosher and Lu, 2020). These lasting improvements are known as perceptual learning.

Perceptual learning is often highly specific, with improvements being tied to a particular stimulus or the area of the visual field where the training occurred. For example, if training takes place in the upper-left visual field, performance is better in this location than in others (Karni and Sagi, 1991). In some instances, however, learning does generalize to new locations, despite remaining strongest at the location where the training occurred. Understanding why learning sometimes transfers to new locations, while remaining localized in other cases, is a central challenge in vision science.

One leading theory proposes that perceptual learning arises through a process known as selective reweighting, in which the brain gradually optimizes how it processes visual information through repeated experiences (Dosher and Lu, 2020). According to this idea, each trial slightly adjusts how different sources of information are weighted during decision making (Petrov et al., 2005). Thus, what a person sees in one trial can influence how they respond in subsequent trials, which may lead to serial dependence. Now, in eLife, Nagu Pinchuk-Yacobi (Bar-Ilan University), Dov Sagi (Weizmann Institute of Science) and Yoram Bonneh (Bar-Ilan) report new insights into the relationship between perceptual learning and serial dependence (Pinchuk-Yacobi et al., 2026).

Serial dependence is a phenomenon in which current perception is biased toward what was seen recently. Pinchuk-Yacobi et al. investigated whether this form of short-term memory is related to the ability to transfer perceptual learning to new visual locations. They reanalysed over 200,000 trials from a large perceptual learning study employing classic texture-discrimination tasks (Harris et al., 2012). In these experiments, observers focused on a central letter while also judging the orientation of a target pattern composed of three oblique lines, which was embedded in a background of horizontal lines (Figure 1).

**Figure 1.**
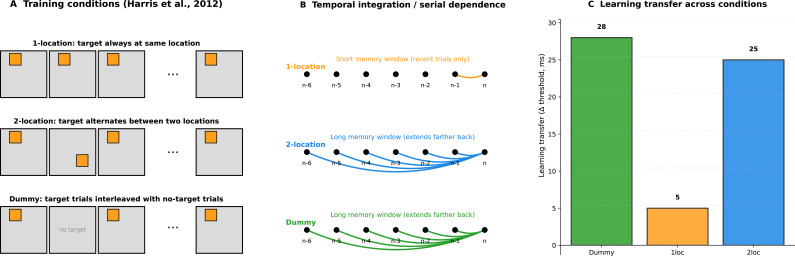
Longer temporal integration promotes generalization in perceptual learning. (**A**) In the 1-location training condition (top), the target always appeared at the same position. In the 2-location condition (middle), it alternated between two positions. In the dummy condition (bottom), the target was present in some trials but not in others. (**B**) Schematic illustration of temporal integration (serial dependence), showing how previous trials influence the current trial. In the 1-location condition (orange), this influence is limited to recent trials, reflecting a short integration window. In the 2-location condition (blue) and the dummy condition (green), this influence extends further back in trial history (longer integration window). (**C**) Learning transfer, measured as the change in performance threshold (Δ threshold, in milliseconds) during a later transfer phase. Larger values indicate greater improvement at a new, untrained location. Robust transfer was observed after dummy (green) and 2-location (blue) training, but not after 1-location training (orange).

Pinchuk-Yacobi et al. compared three training conditions known to produce different levels of transfer. In the 1-location condition, the target always appeared at the same position; in the 2-location condition, the target alternated between two positions; and in the ‘dummy condition’, target-present trials were intermixed with target-absent trials. The researchers then quantified the serial dependence effect by measuring how stimuli from as many as 10 previous trials influenced responses in the current trial.

A striking pattern emerged. In conditions that promoted transfer (the 2-location condition and the dummy condition), stimuli from four to six trials earlier strongly biased current perception. Conversely, this long-range effect was weaker in the 1-location condition. Across participants, stronger long-range serial dependence predicted greater transfer of learning to new locations. However, when just the most recent trial influences the current trial, transfer did not occur. These findings suggest that when target locations are fixed and predictable, as in the 1-location condition, learning tends to become more location-specific, with limited transfer to new locations. When locations vary across trials, as in the 2-location and dummy conditions, however, information is integrated over longer timescales, allowing the visual system to filter out location-specific noise and extract more generalizable features, supporting greater transfer of learning.

Learning transfer is influenced by various factors, including adaptation, task difficulty, attention and training structure (Lu and Dosher, 2022). The study of Pinchuk-Yacobi et al. links serial dependence to adaptation-modulated transfer. Rather than simply being a byproduct of perception, serial dependence may reflect the ongoing adjustments through which perceptual learning occurs. When the influence of past trials extends further back in time, the system is better equipped to transfer skills beyond specific training conditions.

A critical next step will be to determine how other training variables influence serial dependence: specifically, do these other variables extend or shorten the influence of past experiences? More broadly, this framework links short-term memory biases studied in cognitive psychology with long-term neural plasticity studied in neuroscience, offering a unified perspective that could inform fields ranging from reading and education to sports training.
